# PneumoScore: Risk Prediction Model for 90-Day Mortality After Lung Resection

**DOI:** 10.1245/s10434-026-19668-0

**Published:** 2026-05-07

**Authors:** Renata Matheus Faccioli, Luisa Mendes Heise, Leticia Leone Lauricella, Paulo Manuel Pêgo-Fernandes, Ricardo Mingarini Terra

**Affiliations:** 1 Department of Thoracic Surgery, Clinics Hospital, University of São Paulo Medical School, São Paulo, Brazil; 2 University of São Paulo Polytechnic School, São Paulo, Brazil; 3 University os São Paulo Medical School, São Paulo, Brazil

**Keywords:** Lung cancer, Lung resection, Risk prediction, 90-day mortality, Machine learning

## Abstract

**Background:**

Lung resection is the gold-standard treatment for early stage lung cancer, but remains associated with significant mortality, highlighting the need for accurate preoperative risk prediction. This study developed a novel model to predict 90-day mortality after lung cancer resection and compared its performance with well-established models.

**Methods:**

Data were extracted from the Brazilian Lung Cancer Registry, a multicentric database including 2885 patients who underwent lung resection between 2002 and 2024. Records missing more than 15% of variables or any essential variable were excluded. The dataset was randomly divided into training (70%) and testing (30%) subsets. Candidate predictors were selected on the basis of clinical relevance and univariable analyses and multiple machine learning algorithms were evaluated. Final performance was assessed by area under receiver operating characteristic curve (AUROC), area under precision-recall curve (AUPR), Brier Score, Brier Skill Score (BSS), calibration slope, and intercept, using nonparametric bootstrapping to quantify variability.

**Results:**

In total, 2001 patients were included, of whom 88 (4.40%) died within 90 days post surgery. The best-performing model, PneumoScore, was a logistic regression incorporating age, sex, predicted postoperative FEV1%, ASA ≥ IV, coronary artery disease, cerebrovascular disease, congestive heart failure, pneumectomy, thoracotomy, and extended resection. PneumoScore achieved an AUROC of 0.84 (0.77–0.91), AUPR of 0.99 (0.98–1.00), Brier Score of 0.033 (0.022–0.045), BSS of 0.256 (0.180–0.332), intercept of 0.067 (−0.045, 0.179), and slope of 1.067 (0.912–1.222).

**Conclusions:**

PneumoScore demonstrated excellent performance for predicting 90-day mortality, a critical yet underexplored outcome in thoracic surgery, supporting its potential to enhance preoperative evaluation of patients with lung cancer.

**Supplementary Information:**

The online version contains supplementary material available at 10.1245/s10434-026-19668-0.

Risk prediction models have emerged across medical specialties as auxiliary tools for interpreting clinical data and making therapeutic decisions, particularly by estimating the risk of adverse outcomes based on individual patient characteristics.^1–3^ In thoracic oncology, these models primarily focus on the morbidity and mortality associated with lung cancer treatment.

Currently, lung resection with adequate margins and lymph node dissection is the gold standard for early stage lung cancer,^4^ with outcomes significantly improved by minimally invasive techniques.^5–7^ Nonetheless, lung resection remains associated with significant morbidity and mortality,^7^ while various effective nonsurgical treatments such as stereotactic body radiation therapy have emerged.^8^ Thus, the importance of adequately evaluating patients and referring high-risk cases to therapeutic alternatives is evidenced.

Mortality risk prediction models are increasingly used in this context.^9^ Well-established models, such as Thoracoscore,^10^ Modified Thoracoscore,^11^ European Society Objective Score,^12^ Brunelli,^13^ Eurolung2,^14^ and Modified Eurolung2,^15^ have demonstrated significant results, leading to recommendations in international guidelines.^16,17^ However, recent studies highlight their clinical limitations, stemming from factors such as outdated datasets and insufficient predictive variables.^18^ With ongoing technological advancements, improved surgical outcomes, and evolving patient demographics, these models are undergoing continuous performance decline.

In this context, artificial intelligence emerges as a promising tool. With ongoing digitalization of healthcare and expansion of high-quality clinical databases, machine learning techniques stand out for their capacity to refine analyses and continuously improve predictive algorithms.^19–21^ Considering the need for updated and dynamic risk prediction tools in modern thoracic surgery, this study investigates the potential of machine learning to develop a novel model aimed at enhancing preoperative assessment and clinical decision-making for patients with lung cancer.

The primary objective of this study is to develop a model to predict 90-day mortality following lung cancer resection. As a secondary objective, the model’s performance will be evaluated and compared with that of well-established thoracic surgery risk prediction tools, used as benchmarks.

## Methods

The development and evaluation of the risk prediction model followed the Transparent Reporting of a Multivariable Prediction Model for Individual Prognosis or Diagnosis guidelines.^22^ This study was approved by the ethics committee of the University of Sao Paulo Medical School, and all participating institutions and informed consent was obtained from all participants included in the study. Owing to ethical and institutional privacy restrictions, data and analytical code are not publicly available, but access may be granted upon reasonable request and approval by the coordinating center and ethical review boards. There was no patient or public involvement in this study. Statistical analyses and model development were performed using Scikit-learn,^23^ Lifelines,^24^ and NumPy.^25^

Retrospective data were extracted from the Brazilian Lung Cancer Registry (RBCP), a multicentric database comprising patients with lung cancer who underwent lung resection with curative intent.^26^ Currently, the RBCP includes 17 clinical institutions, encompassing public and private hospitals of tertiary and quaternary level. Patient data are retrospectively entered and stored in RedCap, with quality control tools that enable periodic or real-time indirect audits. Procedures include wedge resection, segmentectomy, lobectomy, bilobectomy, and pneumonectomy, performed via thoracotomy, video-assisted thoracic surgery (VATS), or robotic-assisted thoracic surgery (RATS).

The database comprised data from 2885 patients who underwent lung resection between March 2002 and August 2024, of whom 2001 were included in this study. Inclusion criteria encompassed all patients with lung cancer who underwent lung resection with curative intent. Exclusion criteria were applied for records missing more than 15% of variables or missing any essential variables—“sex,” “age,” “planned lung resection,” “planned access,” “status at discharge,” “status at 30 days,” “status at 90 days,” and “overall survival.” For the records included, missing data were addressed using deterministic imputation, whereas the median was applied for numerical variables and the mode for categorical variables when this was not feasible. Although advanced imputation methods such as missForest or MICE have been shown to yield improved performance in certain settings^27^ these gains are often modest and come at the cost of increased computational demand and complexity. In similar contexts, simpler imputation techniques performed comparably when missingness was limited.^28^ Importantly, imputation parameters were derived exclusively from the training partition and subsequently applied to the testing partition, preventing information leakage.

The primary outcome for the machine learning model was all-cause mortality within 90 days following lung resection. The selection of this endpoint, differing from most models which predict in-hospital or 30-day mortality, is supported by strong evidence that 90-day mortality provides a more comprehensive assessment of short-term postoperative risk, capturing a substantial proportion of events that occur between 31 and 90 days post surgery.^29–33^

The dataset was randomly divided into training (70% of data) and testing (30% of data) subsets. The training set was used for model development, while the testing set was used exclusively for final model evaluation, minimizing the risk of overfitting and providing more generalizable performance metrics. Given the event rate, we intentionally preserved the empirical class distribution without applying oversampling or synthetic generation methods such as SMOTE, as these approaches have been shown to distort probability estimates and harm calibration in clinical risk models.^34–36^ Internal validation was strengthened through nonparametric bootstrapping to quantify variability in discrimination and calibration metrics.

A total of 110 variables were collected and analyzed, with the most relevant encompassing demographic characteristics (age, sex, and race), preoperative clinical status (smoking status, comorbidities, anthropometric measurements, spirometric parameters, and symptom and performance scores), oncologic disease characteristics (disease timeline, clinical manifestations, imaging findings, diagnostic workup, histologic classification, detailed staging, tumor location, and adjacent invasion), surgical approach (type of resection, type of surgical access, extent and anatomical location of resection, and additional procedures), and postoperative outcomes (complications, in-hospital mortality, 30-day mortality, and 90-day mortality). Race was categorized as white, Brown, Black, Asian, or Indigenous, on the basis ofself-identification according to the classification system of the Brazilian Institute of Geography and Statistics (IBGE).^37^

Predictor selection was restricted to preoperative or plannable variables to maximize clinical applicability. For variables related to surgical access and extent of resection, both the planned and the performed procedure were recorded. For model development, these variables were defined according to the surgical access and resection actually performed, as postoperative outcomes are more directly related to the surgical exposure ultimately experienced by the patient. However, for prospective application of the model, the values entered should correspond to the planned access and extent of resection, as this reflects the information available at the time of preoperative assessment.

Candidate predictors were identified based on a combination of established clinical relevance and univariable analyses using a liberal threshold (*p* < 0.150), to avoid prematurely excluding potentially important covariates. More complex data-driven selection techniques such as stepwise procedures, penalized regression, or automated feature elimination were not employed, as these may prioritize statistical fit over clinical plausibility and inadvertently retain spurious associations that may limit generalizability. By prioritizing clinically validated predictors, we aimed to ensure causal interpretability, reduce overfitting, and enhance the model’s credibility and utility in perioperative practice.

Several supervised learning algorithms were evaluated, including logistic regression, random Forest, support vector machine, XGBoost, and a simple ensemble, using fivefold cross-validation within the training partition. Final model performance was evaluated on the testing dataset, using nonparametric bootstrapping to quantify variability of metrics. The final model output was presented as probability of death within 90 days of lung resection. Discrimination was assessed using area under the receiver operating characteristic curve (AUROC) and area under the precision-recall curve (AUPR), while calibration was assessed using Brier Score, Brier Skill Score, calibration slope, and calibration intercept.

Benchmark models—Thoracoscore,^10^ Modified Thoracoscore,^11^ European Society Objective Score,^12^ Brunelli,^13^ Eurolung2,^14^ and Modified Eurolung2^15^—were applied following their original formulations. Because these comparators were designed for different endpoints, namely in-hospital and 30-day mortality, their inclusion serves as contextual references rather than formal comparison.

## Results

The dataset included records from 2001 patients, regarding 110 variables. Mortality rates were 2.35% in-hospital, 3.15% within 30 days post surgery, and 4.40% within 90 days post surgery. Postoperative complications occurred in 38.03% of patients. Survival, defined as the period between date of surgery and date of death or censoring, had a mean of 36.72 months and a median of 27.43 months. Table 1 summarizes postoperative outcomes, while Table 2 provides a descriptive and comparative analysis of the main variables in RBCP, indicating statistical significance regarding 90-day mortality.

**Table 1:** Postoperative outcomes of the RBCP

	*N*	%
Mortality		
In-hospital	47	2.35%
30-day mortality	63	3.15%
90-day mortality	88	4.40%
Postoperative complications		
None	1240	61.97%
Pneumonia	159	7.95%
Air leak > 5 days	163	8.15%
Subcutaneous emphysema	140	7.00%
Atelectasis	93	4.65%
Constipation	91	4.55%
Decompensated chronic obstructive pulmonary disease	76	3.80%
Atrial arrhythmia	73	3.65%
Sepsis	63	3.15%
Pneumothorax	59	2.95%
Pleural effusion	57	2.85%

	Mean	SD
Survival	36.72	40.73

Categorical variables are presented as frequencies (n) and percentages (%), while numerical variables are expressed as mean ± standard deviation (SD)

**Table 2:** Descriptive analysis and statistical significance of the main variables of RBCP, considering postoperative mortality within 90-days

	Total		Alive		Dead		*p*-Value
	*N*	%	*N*	%	*N*	%	
Sex^a^							< 0.001
Female	1129	56.42%	1101	57.55%	28	31.82%	
Male	872	43.58%	812	42.45%	60	68.18%	
Race							< 0.592
White	1494	74.66%	1423	78.10%	17	80.68%	
Brown	210	10.49%	204	10.98%	6	6.82%	
Black	88	4.40%	83	4.60%	5	5.68%	
Asian	55	2.75%	54	2.88%	1	1.14%	
Indigenous	1	0.05%	1	0.05%	0	0.00%	
Smoking status^a^							< 0.001
Ex-smoker	930	46.48%	884	46.21%	46	52.27%	
Non-smoker	558	27.89%	551	28.80%	7	7.95%	
Current smoker	399	19.94%	372	19.45%	27	30.68%	
ASA^a^							< 0.001
I	134	6.70%	133	6.95%	1	1.14%	
II	1369	68.42%	1323	69.16%	46	52.27%	
III	397	19.84%	364	19.03%	33	37.50%	
IV	14	0.70%	9	0.47%	5	5.68%	
ECOG^a^							< 0.001
0	1172	58.57%	1149	60.06%	23	26.14%	
1	553	27.64%	514	26.87%	39	44.32%	
2	57	2.85%	49	2.56%	8	9.09%	
3	6	0.30%	3	0.16%	3	3.41%	
MRC^a^							< 0.001
0	1074	53.67%	1045	54.63%	29	32.95%	
1	352	17.59%	325	16.99%	27	30.68%	
2	127	6.35%	118	6.17%	9	10.23%	
3	20	1.00%	17	0.89%	3	3.41%	
4	4	0.20%	3	0.16%	1	1.14%	
Cardiac comorbidities							
None^**a**^	807	40.33%	785	41.04%	22	25.00%	0.004
Hypertension	1006	50.27%	957	50.03%	49	55.68%	0.353
Coronary artery disease	172	8.60%	161	8.42%	11	12.50%	0.253
Arrhythmia^**a**^	65	3.25%	58	3.03%	7	7.95%	0.025
Congestive heart failure^**a**^	49	2.45%	31	1.62%	18	20.45%	< 0.001
Previous cardiac surgery	17	0.85%	14	0.73%	3	3.41%	0.037
Other comorbidities							
None	173	8.65%	169	8.83%	4	4.55%	0.228
Chronic obstructive pulmonary disease^**a**^	702	35.08%	653	34.13%	49	55.68%	< 0.001
Diabetes mellitus	423	21.14%	400	20.91%	23	26.14%	0.298
Hyperlipidemia	295	14.74%	284	14.85%	11	12.50%	0.650
Alcoholism	91	4.55%	85	4.44%	6	6.82%	0.433
Peripheral vascular disease	71	3.55%	69	3.61%	2	2.27%	0.714
Cerebrovascular disease^**a**^	59	2.95%	52	2.72%	7	7.95%	0.012
Connective tissue disease	57	2.85%	53	2.77%	4	4.55%	0.515
Chronic kidney disease^**a**^	56	2.80%	47	2.46%	9	10.23%	< 0.001
Clinical TNM							0.062
IA1	163	8.15%	162	8.47%	1	1.14%	
IA2	415	20.74%	403	21.07%	12	13.64%	
IA3	309	15.44%	294	15.37%	15	17.05%	
IB	147	7.35%	140	7.32%	7	7.95%	
IIA	178	8.90%	168	8.78%	10	11.36%	
IIB	239	11.94%	229	11.97%	10	11.36%	
IIIA	236	11.79%	219	11.45%	17	19.32%	
Type of access^a^							< 0.001
Thoracotomy	738	36.88%	677	35.39%	61	69.32%	
VATS	898	44.88%	876	45.79%	22	25.00%	
RATS	365	18.24%	360	18.82%	5	5.68%	
Type of resection^a^							< 0.001
Wedge resection	57	2.85%	55	2.88%	2	2.27%	
Segmentectomy	174	8.70%	172	8.99%	2	2.27%	
Lobectomy	1627	81.31%	1559	81.50%	68	77.27%	
Bilobectomy	60	3.00%	55	2.88%	5	5.68%	
Pneumonectomy	83	4.15%	72	3.76%	11	12.50%	
Extended resection							0.126
Yes	172	8.60%	160	8.36%	12	13.64%	

	Total		Alive		Dead		*p*-Value
	Mean	SD	Mean	SD	Mean	SD	
Age (years)^a^							< 0.001
	64.05	*11.82*	63.68	*11.83*	72.13	8.17	
BMI (kg/m^2^)							0.070
	26.69	*5.21*	26.74	*5.22*	25.70	*4.88*	
ppoFEV1%^a^							0.017
	66.70	*16.67*	66.94	*16.59*	61.41	*17.76*	
Charlson^a^							< 0.001
	4.86	*1.73*	4.79	*1.71*	6.20	*1.74*	

Categorical variables are presented as frequencies (n) and percentages (%), while numerical variables are expressed as mean ± standard deviation

ASA, American Society of Anesthesiologists Classification; BMI, body mass index; Charlson, Charlson Comorbidity Index Score; ECOG, Eastern Cooperative Oncology Group Performance Status; MRC, Medical Research Council Dyspnoea Scale; ppoFEV1%, predicted postoperative forced expiratory volume in 1 second; RATS, robotic-assisted thoracic surgery; VATS, video-assisted thoracic surgery

^a^Statistically significant variables, defined by *p*-value < 0.05

Several supervised learning algorithms were evaluated, and, despite the inclusion of nonlinear classifiers, the logistic regression model consistently achieved the best balance between discrimination, calibration, and fold-to-fold stability. Using fivefold cross-validation within the training partition, it achieved an AUROC of (0.808 ± 0.138), outperforming the ensemble (0.772 ± 0.109), random Forest (0.736 ± 0.084), XGBoost (0.700 ± 0.124), and support vector machine (0.570 ± 0.209). Owing to its superior performance in the training partition, combined with greater interpretability and ease of integration into perioperative workflows, logistic regression was selected as the final model specification. The final model, designated PneumoScore, utilized the following formula, with binary variables coded 0 for absence and 1 for presence:

$${\mathrm{Logit}}\left( p \right) = - {8}.{24}0{3} + \left( {0.0{761} \times {\mathrm{Age}}} \right) - \left( {0.0{145 } \times {\text{ Predicted}}\;{\mathrm{postoperative}}\;{\mathrm{FEV1}}\% } \right){-}\left( {0.{3721 } \times {\text{ Female}}\;{\mathrm{sex}}} \right) + \left( {0.{1151} \times {\mathrm{Coronary}}\;{\mathrm{artery}}\;{\mathrm{disease}}} \right) + \left( {0.{1418} \times {\mathrm{Cerebrovascular}}\;{\mathrm{disease}}} \right) + \left( {{1}.{7622} \times {\mathrm{Congestive}}\;{\mathrm{heart}}\;{\mathrm{failure}}} \right) + \left( {{1}.0{187} \times ASA \ge 4} \right) + \left( {0.{2171} \times {\mathrm{Extended}}\;{\mathrm{resection}}} \right) + \left( {{1}.{1168} \times {\mathrm{Pneumectomy}}} \right) + \left( {{1}.{5244} \times {\mathrm{Thoracotomy}}} \right)$$

Table 3 compares PneumoScore and the benchmark models, considering the predicting variables and the primary outcome of each model.

**Table 3:** Comparison of predicting variables and primary outcome of PneumoScore and benchmark model*s*

	PneumoScore	Thoracoscore	Modified thoracoscore	ESOS	Brunelli	EuroLung2	Modified EuroLung2
Predictors							
Age							
*p*-value < 0.001	X	X	X	X	X	X	X
Male sex							
*p*-value < 0.001	X	X	X			X	X
ppoFEV1%							
*p*-value 0.017	X			X	X	X	X
ASA							
*p*-value < 0.001	X	X	X				
ECOG							
*p*-value < 0.001		X	X				
MRC							
*p*-value < 0.001		X					
BMI							
*p*-value 0.070						X	X
Charlson							
*p*-value < 0.001		X	X				
Any cardiac comorbidity							
*p*-value 0.004					X		
Coronary artery disease							
*p*-value 0.253	X					X	
Congestive heart failure							
*p*-value < 0.001	X						
Cerebrovascular disease							
*p*-value 0.012	X				X	X	
Malignancy							
*p*-value > 0.999		X	X				
Pneumectomy							
*p*-value < 0.001	X	X	X			X	X
Thoracotomy							
*p*-value < 0.001	X					X	X
Extended resection							
*p*-value 0.126	X				X	X	
Urgency or emergency							
*p*-value > 0.999		X	X				
Primary outcome							
Mortality	90-day	In-hospital	In-hospital	In-hospital	30-day	30-day	30-day

ASA, American Society of Anesthesiologists Classification; BMI, body mass index; Charlson, Charlson Comorbidity Index Score; ECOG, Eastern Cooperative Oncology Group Performance Status; ESOS, European Society Objective Score; MRC, Medical Research Council Dyspnoea Scale; ppoFEV1%, predicted postoperative forced expiratory volume in 1 second

**Fig. 1:**
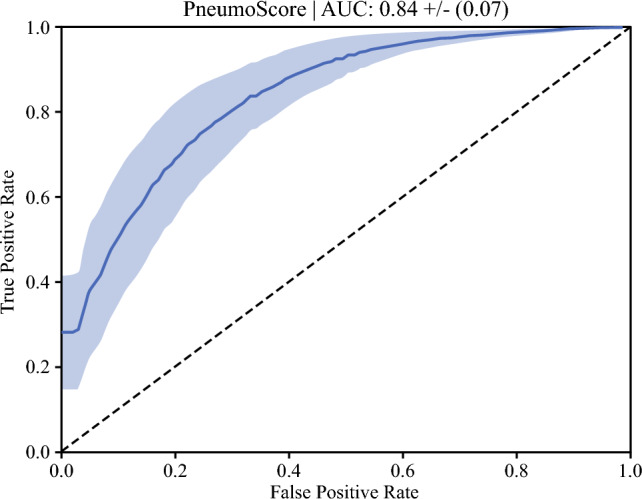
Receiver operating characteristic curve for PneumoScore

**Fig. 2:**
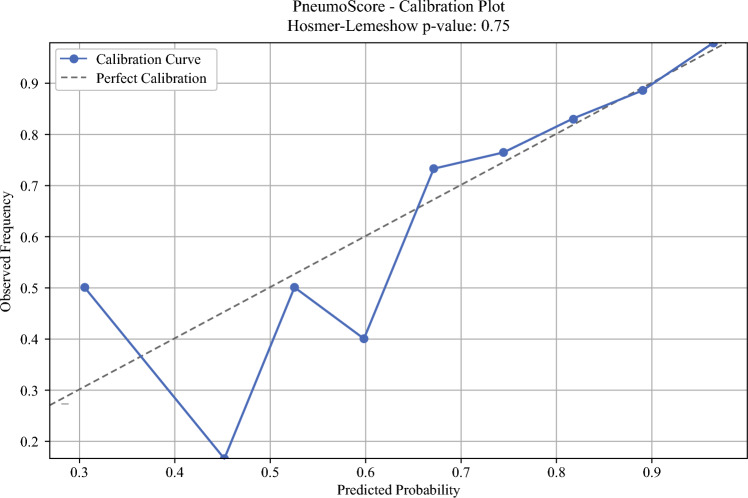
Calibration curve for PneumoScore

Final prediction performance for PneumoScore and the benchmark models was evaluated on the testing dataset according to each model’s primary outcome, with all performance metrics summarized in Tables 4 and 5. The ROC and calibration curves for PneumoScore are presented in Figs. 1 and 2, and the curves for each benchmark model are available in the Supplementary Material. PneumoScore demonstrated excellent discriminative performance, with an AUROC of 0.84 (0.77–0.91) and an AUPR of 0.99 (0.98–1.00), and strong calibration, with an intercept of 0.067 (−0.045 to 0.179), a calibration slope of 1.067 (0.912–1.222), a Brier Score of 0.033 (0.022–0.045) and a Brier Skill Score of 0.256 (0.180–0.332).

**Table 4:** Discrimination metrics of all risk prediction models in the testing dataset, with 95% confidence interval

	AUROC	AUPR
PneumoScore	0.84 (0.77–0.91)	0.99 (0.98–1.00)
Thoracoscore	0.78 (0.66–0.90)	0.12 (0.02–0.34)
Modified thoracoscore	0.79 (0.68–0.90)	0.13 (0.02–0.36)
European society objective score	0.70 (0.56–0.84)	0.07 (0.01–0.20)
Brunelli	0.74 (0.65–0.83)	0.12 (0.04–0.26)
EuroLung2	0.79 (0.73–0.85)	0.10 (0.05–0.20)
Modified EuroLung2	0.78 (0.70–0.86)	0.09 (0.04 - 0.17)

AUROC, Area under de receiver operating characteristic curve; AUPR, area under de precision-recall curve

**Table 5:** Calibration metrics of all risk prediction models in the testing dataset, with 95% confidence interval

	Brier score	BSS	Calibration intercept	Calibration slope
PneumoScore	0.033 (0.022–0.045)	0.256 (0.180–0.332)	0.067 (−0.045 to 0.179)	1.067 (0.912–1.222)
Thoracoscore	0.016 (0.007–0.026)	0.016 (−0.052 to 0.063)	1.474 (−1.824 to 5.407)	1.489 (0.668–2.604)
Modified thoracoscore	0.018 (0.009–0.029)	−0.131 (−0.603 to 0.180)	−1.503 (−3.172 to 0.355)	0.622 (0.314–1.100)
European society objective score	0.017 (0.007–0.028)	0.005 (−0.024 to 0.025)	1.215 (−3.939 to 6.407)	1.396 (0.123–2.894)
Brunelli	0.028 (0.017–0.039)	-0.003 (−0.111 to 0.081)	−1.176 (−2.576 to 0.075)	0.680 (0.317–1.094)
EuroLung2	0.028 (0.016–0.040)	0.013 (−0.002–0.032)	1.618 (−0.201 to 3.586)	1.304 (0.858–1.821)
Modified EuroLung2	0.028 (0.016–0.042)	0.005 (−0.009 to 0.018)	1.790 (−0.388 to 4.126)	1.261 (0.756–1.869)

BSS, Brier skill score

## Discussion

This study developed a novel model to predict 90-day mortality after lung resection, aiming to enhance preoperative assessment for patients with lung cancer. PneumoScore demonstrated excellent discriminative performance, with an AUROC of 0.84 (0.77–0.91) and an AUPR of 0.99 (0.98–1.00), and strong calibration, with an intercept (0.067) and slope (1.067) close to ideal values, a low Brier Score (0.033), and high Brier Skill Score (0.256), suggesting accurate and well-calibrated risk estimates across the predicted probability range.

Among the benchmark models, EuroLung2 and Modified EuroLung2 presented good discrimination (AUROC 0.79 and 0.78; AUPR of 0.10 and 0.09) and acceptable calibration, with moderate underestimation of risk (intercept 1.618 and 1.790; slope 1.304 and 1.261) and mild improvement over the null model (BSS of 0.013 and 0.005). Thoracoscore, Modified Thoracoscore, and Brunelli achieved good discrimination (AUROC 0.78, 0.79, and 0.74; AUPR 0.12, 0.13, and 0.12) but poor calibration, with underestimation of risk by Thoracoscore (intercept 1.474; slope 1.489) and overestimation by Modified Thoracoscore and Brunelli (intercept −1.503 and −1.176; slope 0.622 and 0.680) and mild or no improvement over the null model (BSS 0.016, −0.131, 0.003). Finally, European Society Objective Score yielded only acceptable calibration (AUROC 0.70; AUPR 0.07) and unreliable calibration, with extremely wide confidence intervals. Importantly, comparisons between PneumoScore and the benchmark models are inherently limited by the distinct endpoints for which these models were originally developed. To ensure the validity of the performance assessment, each model was evaluated according to its intended outcome, precluding direct statistical comparisons. Accordingly, the inclusion of benchmark models in this study serves primarily to contextualize PneumoScore’s performance relative to existing prognostic tools, rather than to assert formal claims of superiority. Nonetheless, PneumoScore exhibited consistently superior discrimination and calibration within this cohort, supporting its potential value as a contemporary risk prediction model.

One of the primary objectives of this study was to evaluate the potential of different machine learning algorithms for risk prediction in thoracic surgery. Interestingly, logistic regression outperformed more complex models applied to our database. Although unexpected, this observation is consistent with findings reported in other risk prediction studies, likely reflecting the class imbalance inherent to clinical scenarios with relatively low event rates.^38–40^ While advanced techniques such as SMOTE can be used to address class imbalance, they may alter the underlying data distribution and compromise clinical validity and were therefore not applied in this study.^34–36^ These results enable PneumoScore to combine strong predictive performance with greater interpretability and ease of integration into perioperative workflows, underscoring that more sophisticated algorithms do not necessarily yield superior results in every clinical context.

Another important feature of PneumoScore is the use of 90-day mortality as the primary outcome. This time horizon is supported by robust evidence indicating that it provides a more comprehensive assessment of short-term postoperative risk, capturing a substantial proportion of events that occur between 31 and 90 days post surgery.^29–33^ In our dataset, 25 deaths occurred within this period, representing 28% of the total 88 deaths within 90 days post-surgery. Nevertheless, despite its relevance, few thoracic surgery risk prediction models adopt this metric. One notable example is the VATS model,^41^ which demonstrated strong internal validation with a bootstrapped C-index > 0.80 in 92% of samples—however, it is restricted to VATS cases, with a 2.5% 90-day mortality rate, limiting its generalizability. Another example is the National Lung Cancer Audit model,^32^ which was internally and externally validated by O´Dows et al.^42^ with reported AUCs of 0.68 and 0.60, respectively.

Despite the promising results, our study has limitations. As an evolving database, the RBCP presents inherent constraints related to case volume and data completeness. Moreover, while the RBCP reflects the unique healthcare challenges of a developing country, many participating institutions are referral centers equipped to manage high-complexity cases, which may not fully represent the broader Brazilian reality. Consequently, the study cohort included a small but significant number of patients with unfavorable preoperative evaluation, resulting in mortality rates slightly higher than those reported in the literature.^29–33^ Larger and more diverse databases are known to improve the performance of machine learning models, making the ongoing expansion of the RBCP a valuable opportunity for future updates to PneumoScore. While this study focused on the development and internal validation of the model, further external validation is warranted to evaluate the model’s generalizability, both internationally and in Brazilian settings beyond the RBCP.

Additionally, variables with well-established prognostic value, such as diffusing capacity of the lungs for carbon monoxide (DLCO) and predicted postoperative DLCO (ppoDLCO), were excluded due to underrepresentation, as these tests are performed only in selected patients and at specific institutions in our database. While prioritizing variables obtained from universally available tests enhances the model’s applicability across diverse clinical settings, incorporating these and other more specific preoperative metrics could further refine PneumoScore’s predictive performance in future iterations.

Finally, although predictor selection was restricted to preoperative or plannable variables, surgical variables were defined according to the procedure ultimately performed, as these are more directly associated with postoperative outcomes. While this approach improves model performance, it represents a limitation inherent to preoperative risk models, as prospective application relied on the planned procedure, which may differ from the final surgical approach.

In conclusion, PneumoScore demonstrated highly promising results for the preoperative prediction of individual 90-day mortality risk, a critical yet underexplored outcome in thoracic surgery. This study establishes a foundation for positioning PneumoScore as a widely accessible tool to support data interpretation and clinical decision-making in complex cases. To ensure ongoing relevance, periodic updates will be essential to align the model with advances in thoracic surgery, which is facilitated by an already established machine learning pipeline. Furthermore, integration with digital health systems may facilitate its incorporation into routine care with simple data input. Future studies will aim to assess PneumoScore’s generalizability through external validation, as well as further explore its practical applicability and impact on patient outcomes.

## Supplementary Information

Below is the link to the electronic supplementary material.

Supplementary file1 (JPG 31 KB)

Supplementary file2 (JPG 32 KB)

Supplementary file3 (JPG 34 KB)

Supplementary file4 (JPG 30 KB)

Supplementary file5 (JPG 30 KB)

Supplementary file6 (JPG 31 KB)

Supplementary file7 (JPG 59 KB)

Supplementary file8 (JPG 63 KB)

Supplementary file9 (JPG 64 KB)

Supplementary file10 (JPG 64 KB)

Supplementary file11 (JPG 58 KB)

Supplementary file12 (JPG 55 KB)
