# Supplementary figures and images for: PneumoScore: Risk Prediction Model for 90-Day Mortality After Lung Resection
Source: Ann Surg Oncol. 2026 May 7;33(8):7123–32. doi: 10.1245/s10434-026-19668-0 (PMC13337678; doi:10.1245/s10434-026-19668-0)

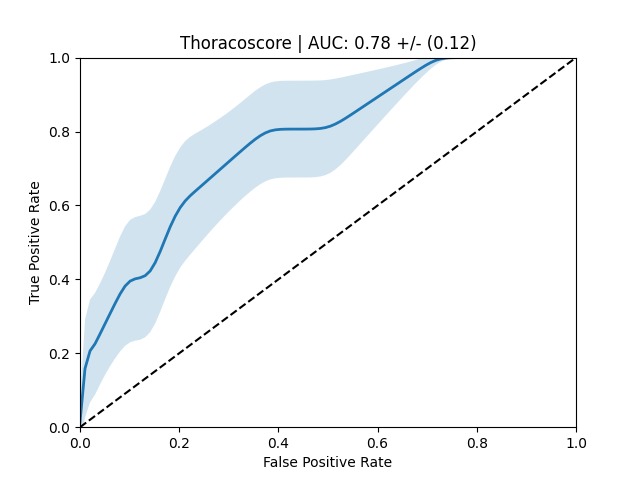

Supplement: Supplementary file 1 — Supplementary file1 (JPG 31 KB) [file 10434_2026_19668_MOESM1_ESM.jpg]

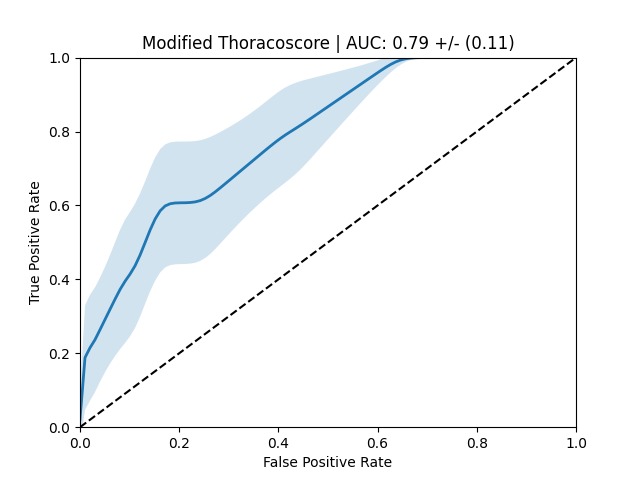

Supplement: Supplementary file 2 — Supplementary file2 (JPG 32 KB) [file 10434_2026_19668_MOESM2_ESM.jpg]

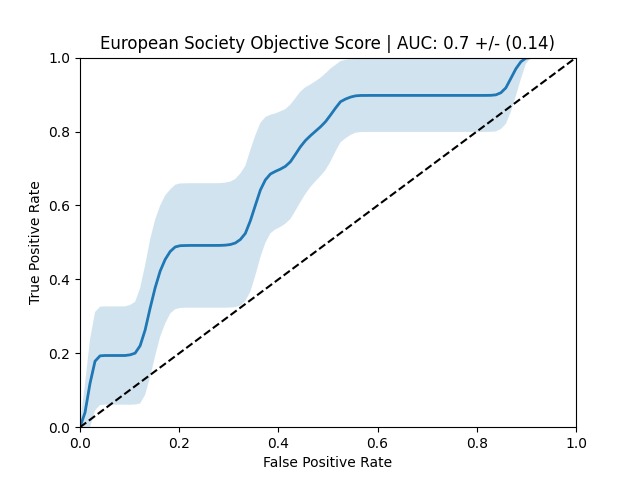

Supplement: Supplementary file 3 — Supplementary file3 (JPG 34 KB) [file 10434_2026_19668_MOESM3_ESM.jpg]

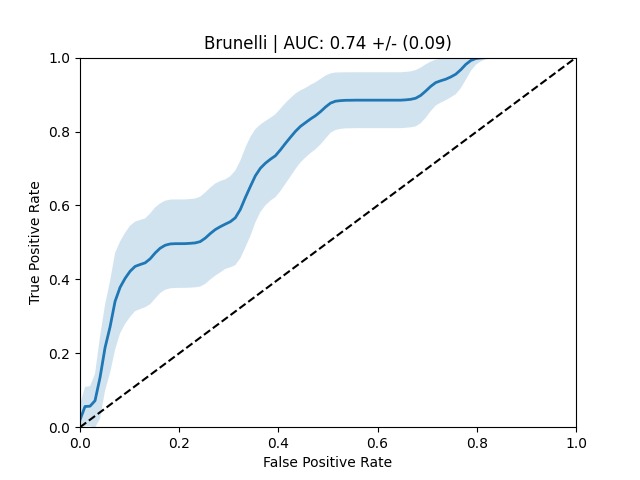

Supplement: Supplementary file 4 — Supplementary file4 (JPG 30 KB) [file 10434_2026_19668_MOESM4_ESM.jpg]

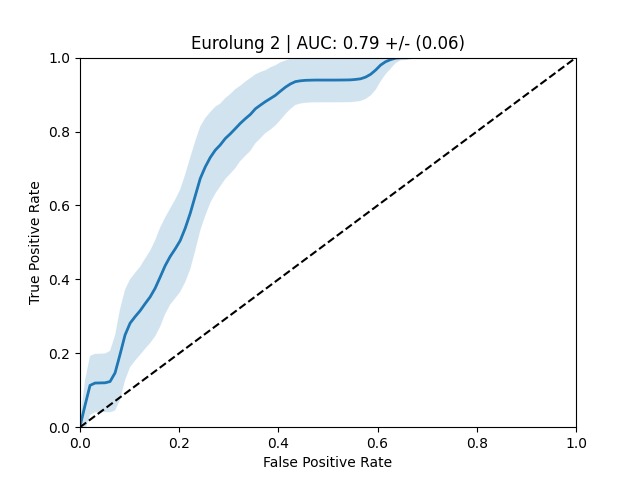

Supplement: Supplementary file 5 — Supplementary file5 (JPG 30 KB) [file 10434_2026_19668_MOESM5_ESM.jpg]

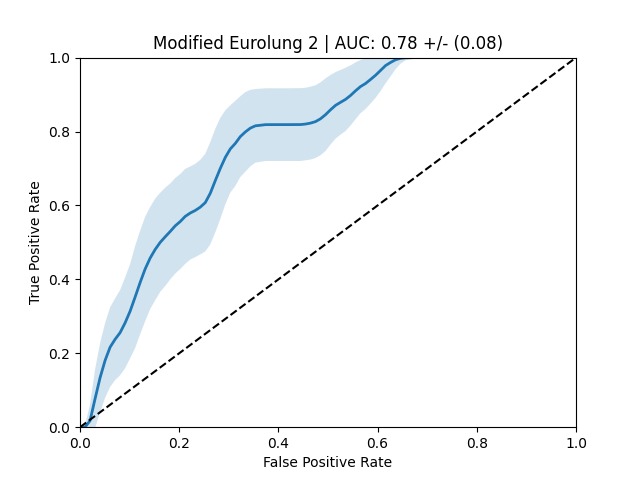

Supplement: Supplementary file 6 — Supplementary file6 (JPG 31 KB) [file 10434_2026_19668_MOESM6_ESM.jpg]

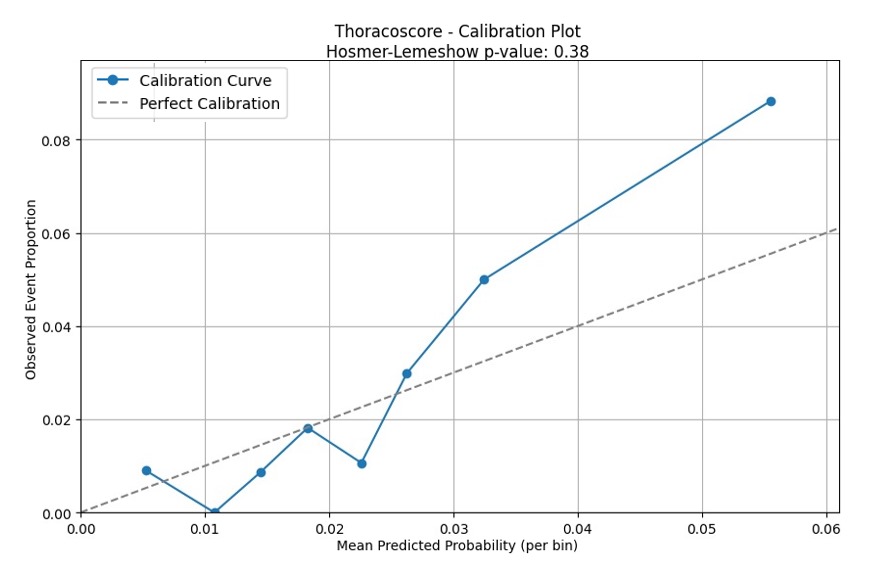

Supplement: Supplementary file 7 — Supplementary file7 (JPG 59 KB) [file 10434_2026_19668_MOESM7_ESM.jpg]

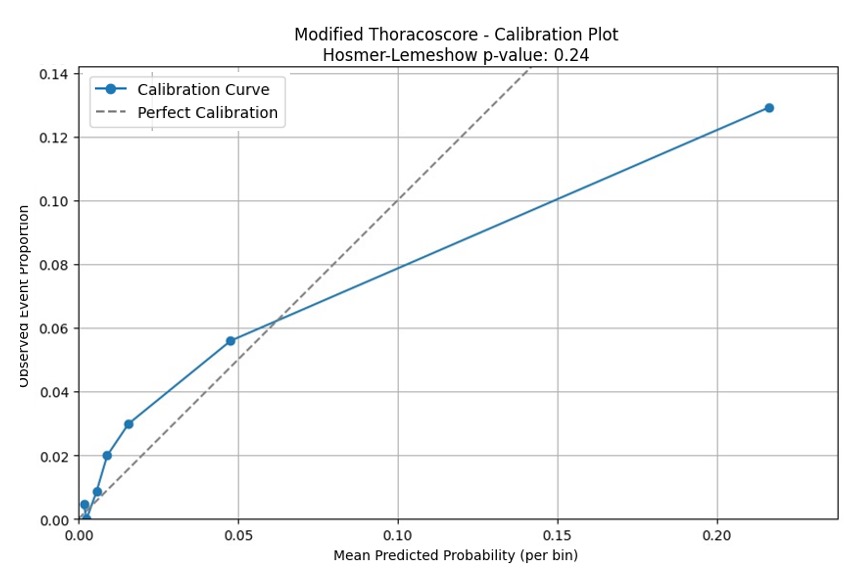

Supplement: Supplementary file 8 — Supplementary file8 (JPG 63 KB) [file 10434_2026_19668_MOESM8_ESM.jpg]

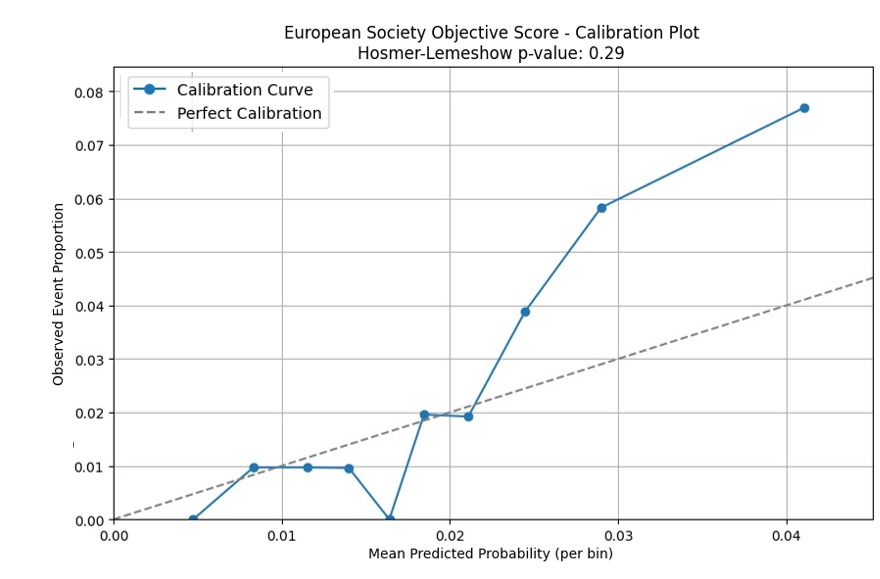

Supplement: Supplementary file 9 — Supplementary file9 (JPG 64 KB) [file 10434_2026_19668_MOESM9_ESM.jpg]

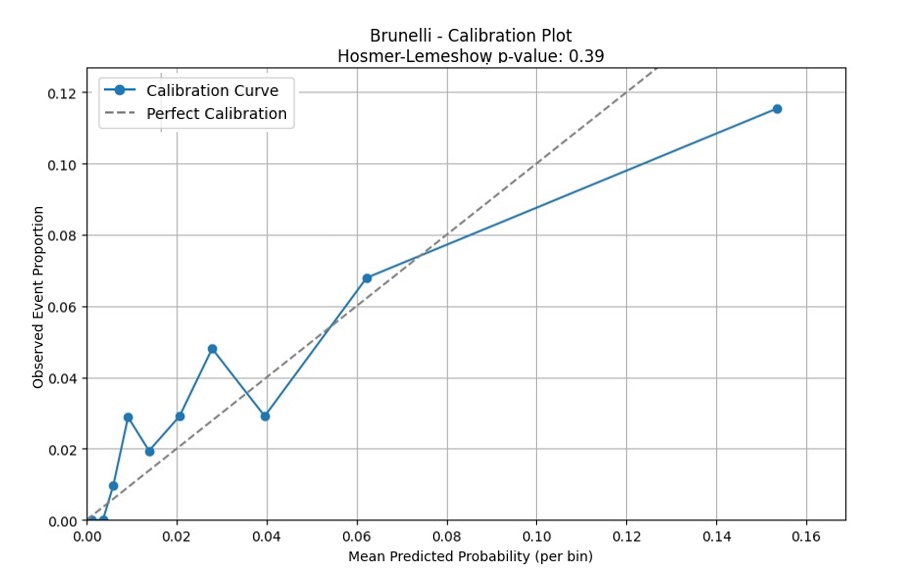

Supplement: Supplementary file 10 — Supplementary file10 (JPG 64 KB) [file 10434_2026_19668_MOESM10_ESM.jpg]

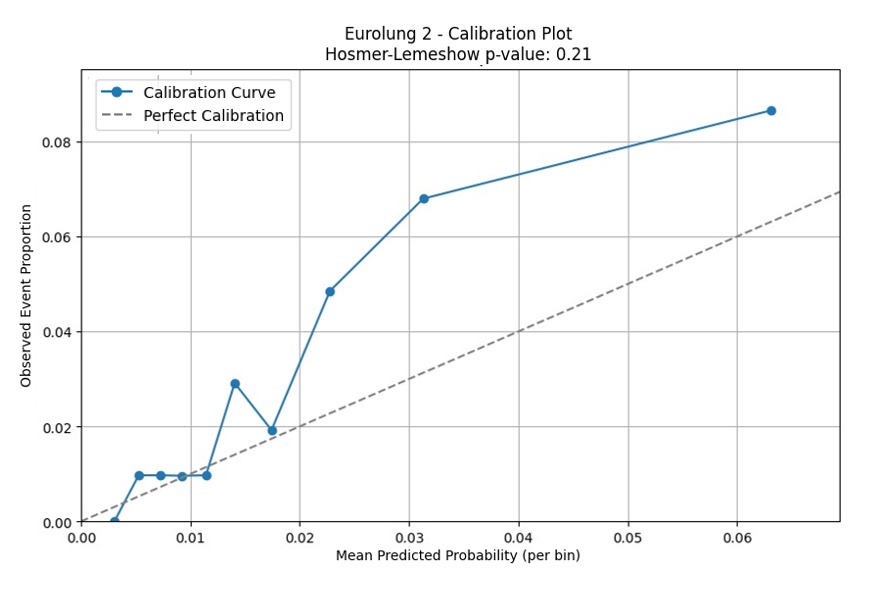

Supplement: Supplementary file 11 — Supplementary file11 (JPG 58 KB) [file 10434_2026_19668_MOESM11_ESM.jpg]

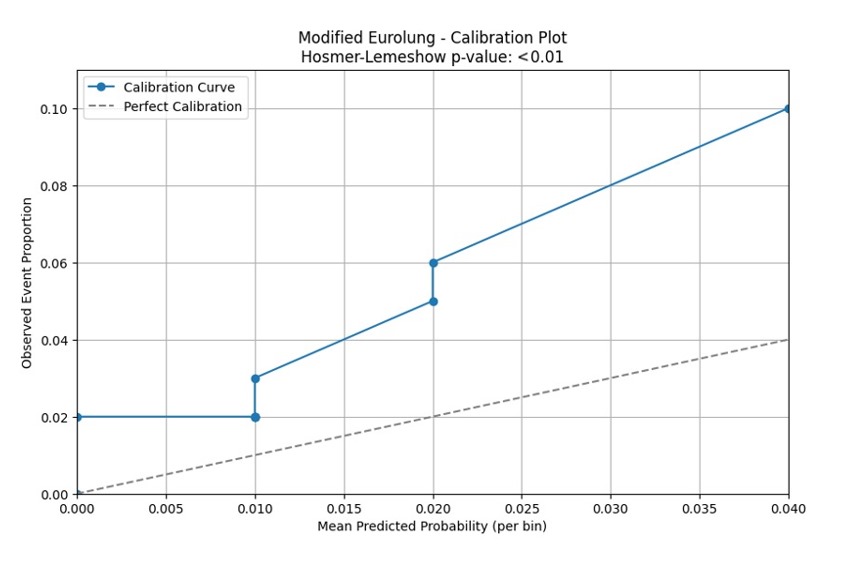

Supplement: Supplementary file 12 — Supplementary file12 (JPG 55 KB) [file 10434_2026_19668_MOESM12_ESM.jpg]
